# RfaH Suppresses Small RNA MicA Inhibition of *fimB* Expression in Escherichia coli K-12

**DOI:** 10.1128/JB.00912-13

**Published:** 2014-01

**Authors:** Alexandra Moores, Saranna Chipper-Keating, Lei Sun, Gareth McVicker, Lynn Wales, Krishna Gashi, Ian C. Blomfield

**Affiliations:** School of Biosciences, University of Kent, Canterbury, Kent, United Kingdom

## Abstract

The phase variation (reversible on-off switching) of the type 1 fimbrial adhesin of Escherichia coli involves a DNA inversion catalyzed by FimB (switching in either direction) or FimE (on-to-off switching). Here, we demonstrate that RfaH activates expression of a FimB-LacZ protein fusion while having a modest inhibitory effect on a comparable *fimB-lacZ* operon construct and on a FimE-LacZ protein fusion, indicating that RfaH selectively controls *fimB* expression at the posttranscriptional level. Further work demonstrates that loss of RfaH enables small RNA (sRNA) MicA inhibition of *fimB* expression even in the absence of exogenous inducing stress. This effect is explained by induction of σ^E^, and hence MicA, in the absence of RfaH. Additional work confirms that the procaine-dependent induction of *micA* requires OmpR, as reported previously (A. Coornaert et al., Mol. Microbiol. 76:467–479, 2010, doi:10.1111/j.1365-2958.2010.07115.x), but also demonstrates that RfaH inhibition of *fimB* transcription is enhanced by procaine independently of OmpR. While the effect of procaine on *fimB* transcription is shown to be independent of RcsB, it was found to require SlyA, another known regulator of *fimB* transcription. These results demonstrate a complex role for RfaH as a regulator of *fimB* expression.

## INTRODUCTION

Like many adhesins, type 1 fimbriation is controlled by phase variation (the reversible on-to-off switching in gene expression that produces a mixed population). *fim* phase variation involves the site-specific inversion of an ∼300-bp promoter element (*fimS*) that contains a promoter for the fimbrial structural genes (1). Inversion is catalyzed by recombinases FimE (on-to-off switching) and FimB (low-frequency switching in either direction), encoded by genes situated adjacent to *fimS* (2), as well as by alternative recombinases situated elsewhere in the chromosome in some pathogenic strains (3, 4).

Regulation of the *fim* inversion is complex and involves changes in both recombinase activity and expression. For example, the availability of the branched-chain amino acids and alanine exerts a direct effect on the *fim* inversion by altering the interaction of the leucine-responsive regulatory protein (Lrp) with the invertible element (5). In contrast, sialic acid and *N*-acetylglucosamine inhibit *fimB* expression, and hence FimB recombination, selectively (6, 7). Moreover, the orientation of *fimS* controls *fimE* expression, ensuring that *fimE* expression is lower in afimbriate than fimbriate cells (8, 9). Type 1 fimbriae are a virulence factor in urinary tract and other infections, and attachment of fimbriate cells is proinflammatory and facilitates intracellular invasion (10–12). We have proposed that, by repressing type 1 fimbrial expression in response to signals like sialic acid, Escherichia coli is better able to avoid host defenses (6).

NusG and its homolog RfaH regulate transcriptional pausing and termination. While NusG has a generalized effect on gene expression and is essential for viability, RfaH is dispensable (13). RfaH controls the expression of a specific subset of genes in E. coli, including those involved in lipopolysaccharide (LPS) core biosynthesis (14–16), as well as virulence factors (17–20). Uropathogenic E. coli (UPEC) mutants lacking *rfaH* are attenuated for virulence in an ascending mouse model of urinary tract infection (21). Remarkably, RfaH has also been shown to stimulate translation by binding to protein S10 of the 30S ribosomal subunit (22). Unlike NusG, both activities of RfaH require the presence of *cis*-acting *ops* (operon polarity suppressor) sites in the DNA that serve to recruit RfaH to a paused RNA polymerase (RNAP) transcription elongation complex (13, 23). The ability of RfaH to switch between transcriptional regulator and translational activator involves an unprecedented refolding of the RfaH carboxy-terminal domain (RfaH-CTD) from an all-α to all-β confirmation, enabling RfaH to bind to ribosomal protein S10 (13).

*fimB*, which has a sigma 70 promoter, has a large (271-bp) 5′ untranslated region (5′ UTR), suggesting that the recombinase gene may be subject to extensive control following transcription initiation (24, 25). While this possibility has yet to be investigated in detail, *fimB* expression has been shown recently to be inhibited by the σ^E^-dependent small RNA (sRNA) MicA (26). Here, we report that *fimB* expression is also enhanced by RfaH and that this effect requires MicA.

## MATERIALS AND METHODS

### Bacterial strains and plasmids.

The bacterial strains used in this work are listed in Table 1. All bacterial strains are derivatives of E. coli K-12. To combine mutant *fimB* alleles with a FimB-LacZ protein fusion, PCR-generated mutant DNAs were first cloned into plasmid pIB347, a derivative of the temperature-sensitive vector pMAK705 (27), to replace the wild-type EcoO109I-SphI (Δ1 mutation) or SphI-ClaI (Δ2 and Δ3, OLE mutations) regions. Likewise, to combine the OLE mutation with the *fimB-lacZ* transcriptional fusion, the mutant DNA was cloned into plasmid pIB342 to replace the SphI-ClaI region. Allelic exchange was then used to transfer the mutations from the plasmids into the chromosomal *fim* region of strain BGEC043 or KCEC840 using *sacB* and sucrose counterselection as described previously (28). A *micA-lacZYA* fusion replacing the wild-type *micA* gene was constructed by inserting an XbaI fragment containing the promoterless *lacZYA* genes isolated from plasmid pIB341 into a *micA* vector (pAM011) to generate pAM012. The *micA-lacZYA* construct was then transferred into the genome by allelic exchange between pAM012 and strain AAEC100 (MG1655 Δ*lacZYA*) (28). Strain AAEC189 (Δ*fim*) was used as the host strain for recombinant plasmids to ensure that the DNA was suitably methylated to allow subsequent transformation of the strain MG1655 (23). P1 transduction was carried out using P1_vir_ as described previously (29).

**TABLE 1:** Strains used in this study

Strain	Relevant characteristics	Source/reference
MG1655	K-12 wild type; λ^−^ F^−^ Fim^+^	E. coli Genetic Stock Center (CGSC) (47)
AAEC189	YMC9 (λ^−^ F^−^ *supE44 hsdR17 mcrA mcrB end A1 thi 1ΔargF-lac-205 ΔfimB-H ΔrecA*	29
JW0052-1	BW25113 Δ*surA*ΩKan^r^	CGSC/Keio collection (48)
JW2205	BW25113 Δ*rcsB*ΩKan^r^	S. Andrews/Keio collection (48)
JW3818	BW25113 Δ*rfaH*ΩKan^r^	National BioResource Project/Keio collection (48)
CAG25198	MG1655 *lacX74* lambda(*rpoH*P3-*lacZ*) *nadB*::Tn*10 ΔrseA*	C. Gross (49)
CAG45114	MG1655 *lacX74* lambda(*rpoH*P3-*lacZ*)	C. Gross (50)
CAG62192	*micA*ΩCam^r^	C. Gross (26)
AAEC090	MG1655 Δ*lacZYA*Ω(*sacB*-Kan^r^)	28
AAEC100	AAEC090 Δ(*sacB*-Kan^r^)	28
AAEC261A	MG1655 Δ*lacZYA fimB-lacZYA*	31
BGEC043	MG1655 Δ*lacZYA* Δ*fimB* (−457 [EcoO109I] to +209 [ClaI] relative to *fimB* ORF) Ω(*sacB*-Kan^r^) *lacZYA*-3' *fimB*	Our unpublished work
BGEC088	MG1655 Δ*lacZYA* FimE-LacZ	6
BGEC378	MG1655 Δ*lacZYA fimA*'-RNase III cleavage site-*lacZYA fimE-am18*	51
BGEC905	MG1655 Δ*lacZYA* FimB-LacZ	6
KCEC840	MG1655 Δ*lacZYA* Δ*fimB* (−1033 [ApaLI] to +209 [ClaI] relative to *fimB* ORF) Ω(*sacB*-Kan^r^) *lacZYA*-3' *fimB*	34
KCEC1243	BGEC905 Δ*slyA*ΩKan^r^	34
KCEC1457	BGEC905 Δ1(Δ*fimB* 5′ UTR −277 bp to −203 bp from *fimB* ORF)	This study
KCEC2862	AAEC261A Δ*slyA*ΩKan^r^	This study
KCEC3700	BGEC905 Δ*rfaH*ΩKan^r^	This study
KCEC3858	BGEC905 Δ3(Δ*fimB* 5′ UTR −196 bp to −20 bp from *fimB* ORF)	This study
KCEC3882	BGEC905 Δ3(Δ*fimB* 5′ UTR −196 bp to −20 bp from *fimB* ORF) Δ*rfaH*ΩKan^r^	This study
KCEC3890	AAEC261A Δ*rfaH*ΩKan^r^	This study
KCEC4138	BGEC905 *micA*ΩCam^r^	This study
KCEC4176	BGEC905 *micA*ΩCam^r^ Δ*rfaH*ΩKan^r^	This study
KCEC4198	BGEC378 Δ*rfaH*ΩKan^r^	This study
KCEC4202	KCEC2862 Δ*slyA*	This study
KCEC4222	KCEC4204 Δ*rfaH*ΩKan^r^	This study
KCEC4271	BGEC905 Rm43 (OLE changed from 5′ GGCGGTAGTto 5′ CCGCTATCA)	This study
KCEC4275	BGEC905 Rm43 (OLE changed from 5′ GGCGGTAGTto 5′ CCGCTATCA) Δ*rfaH*ΩKan^r^	This study
KCEC4279	BGEC905 Rm43 (OLE changed from 5′ GGCGGTAGTto 5′ CCGCTATCA) *micA*ΩCam^r^	This study
KCEC4326	BGEC905 Δ2(Δ*fimB* 5′ UTR −196 bp to −58 bp from *fimB* ORF)	This study
KCEC4336	BGEC905 Δ2(Δ*fimB* 5′ UTR −196 bp to −58 bp from *fimB* ORF) Δ*rfaH*ΩKan^r^	This study
KCEC4364	BGEC905 Δ1(Δ*fimB* 5′ UTR −277 bp to −203 bp from *fimB* ORF) Δ*rfaH*ΩKan^r^	This study
KCEC4370	AAEC261A Rm43 (OLE changed from 5′ GGCGGTAGTto 5′ CCGCTATCA)	This study
KCEC4372	BGEC905 Δ*rfaH*ΩKan^r^ Δ*lacZYA*Ω*rfaH* (from 161 bp upstream to 56 bp downstream of the *rfaH* ORF)	This study
KCEC4386	BGEC088 Δ*rfaH*ΩKan^r^	This study
KCEC4412	CAG45114 Δ*rfaH*ΩKan^r^	This study
KCEC4418	CAG45114 *nadB*::Tn*10* Δ*rseA*	This study
KCEC4420	BGEC905 *nadB*::Tn*10* Δ*rseA*	This study
KCEC4454	AAEC261A Δ*rcsB*ΩKan^r^	This study
KCEC4484	BGEC905 *micA*ΩCam^r^ *nadB*::Tn*10* Δ*rseA*	This study
KCEC4534	AAEC100 *micA-lacZYA*	This study
KCEC4536	KCEC4534 Δ*rfaH*ΩKan^r^	This study
KCEC4540	KCEC4534 *nadB*::Tn*10* Δ*rseA*	This study

### Media and growth conditions.

The media included L broth (5 g of sodium chloride, 5 g of yeast extract, and 10 g of tryptone per liter [Difco]) and L agar (L broth with 1.5% agar [Difco]). Sucrose agar, used to select recombinant bacteria (13), is L agar supplemented with 6% sucrose in the absence of sodium chloride. The antibiotics chloramphenicol (25 μg/ml) and kanamycin (25 μg/ml) were included in selective media as required (Sigma). Lactose MacConkey plates (BD) were used as an indicator medium to determine the proportion of switch-on to switch-off bacteria. For rich defined (RD) medium, minimal MOPS [3-(*N*-morpholino)propanesulfonic acid] medium was prepared as described by Neidhardt et al. (30). The media were supplemented with 10 mM thiamine, 0.4% glycerol, bases, vitamin B supplement, and amino acids as reported originally by Neidhardt et al. (30). In experiments that included an *rseA* mutant control, the medium was supplemented with 1 mM nicotinic acid. All reagents were obtained from Sigma unless otherwise indicated. Liquid cultures were grown aerobically at 37°C, and culture densities were monitored spectrophotometrically at 420 or 600 nm.

### Analysis of *fimB* and *fimE* expression.

*fimB* and *fimE* expression was measured using a FimB-LacZ or FimE-LacZ translational fusion or *fimB-lacZ* transcriptional fusion situated in the chromosome at *fim* as described previously (6, 7, 31). β-Galactosidase assays were conducted as described by us previously (31), following growth in RD medium at 37°C with rapid aeration to an optical density at 600 nm (OD_600_) of approximately 0.2. Experiments were repeated at least twice, and the values shown in Miller units represent the mean of at least four samples with 95% confidence intervals included for each value.

### Determination of inversion frequencies.

Inversion of the *fim* switch was measured following growth in RD medium as described previously (32). Inversion frequencies were measured by inoculating 25 cultures with approximately 0.3 cells per tube. The ratio of on to off cells was estimated by plating at least five replicates onto lactose-MacConkey indicator medium after approximately 22 generations of growth at 37°C with rapid aeration.

### DNA manipulations.

DNA manipulations were carried out using standard protocols (33). Plasmid DNA was isolated using the miniprep or midiprep kit (Qiagen). Restriction enzymes and DNA ligase were purchased from either Promega or New England BioLabs. DNA sequencing was performed by Source BioScience, Nottingham, United Kingdom. Oligonucleotide synthesis was performed by MWG-Biotech AG or by Qiagen Operon, Germany. DNA was amplified by PCR using *Pwo* polymerase (Boehringer Mannheim) as described previously (25) or Q5 master mix (New England BioLabs). Oligonucleotides used in this study are listed in Table S1 in the supplemental material.

## RESULTS

### RfaH is a positive regulator of *fimB* expression.

**Fig 1:**
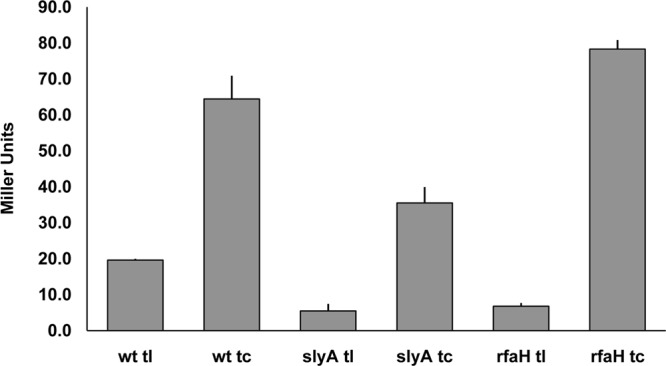
The effects of Δ*rfaH* and Δ*slyA* mutations on the β-galactosidase produced by FimB-LacZ translational (tl) and *fimB-lacZ* transcriptional (tc) fusions. The wild-type (wt) and mutant strains indicated were grown and processed as described in Materials and Methods.

**Fig 2:**
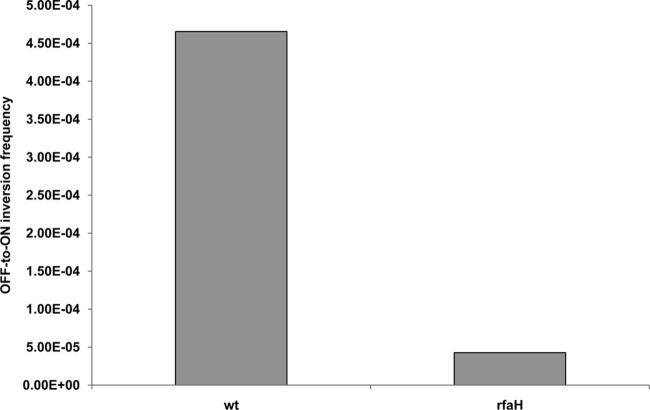
The effects of Δ*rfaH* on FimB off-to-on recombination per cell per generation. The bacteria were grown and processed as described in Materials and Methods. The values shown are the means of at least five measurements.

To determine if RfaH controls *fimB* expression, a Δ*rfaH* mutant of strain BGEC905 (MG1655 Δ*lacZYA* FimB-LacZ) was constructed by P1_vir_ transduction. The level of β-galactosidase produced by the mutant was diminished by almost 3-fold relative to the wild type in this FimB-LacZ protein fusion background (Fig. 1). FimB recombination parallels *fimB* expression closely, and FimB recombination was also diminished around 9-fold in the *rfaH* mutant as anticipated (Fig. 2) (6, 7, 34).

In contrast to the FimB-LacZ fusion, expression of a comparable *fimB-lacZYA* operon fusion increased to a modest extent (∼1.2-fold). Likewise, expression of a FimE-LacZ protein fusion also increased slightly (∼1.2-fold; data not shown). As a control, the Δ*rfaH-fimB* mutant phenotype was complemented by an ectopic copy of the *rfaH* gene inserted into the chromosome at *lac* (data not shown). As an additional control, the effect of SlyA on the β-galactosidase produced by the two fusions was also measured (Fig. 1). SlyA activates *fimB* transcription by inhibiting H-NS repression, and its loss decreased expression of both fusions as expected (34). Thus, RfaH affects *fimB* expression mainly at the posttranscriptional level, to produce a net stimulatory effect on *fimB* expression.

### Identification of a region of the *fimB* 5′ UTR required for RfaH control.

In addition to its effects on transcription termination, RfaH stimulates translation initiation by binding to protein S10 of the 30S ribosomal subunit (22). This suggested to us that RfaH might activate *fimB* translation directly. Alternatively, we supposed that RfaH could activate *fimB* expression indirectly by controlling the expression of posttranscriptional regulator instead.

**Fig 3:**
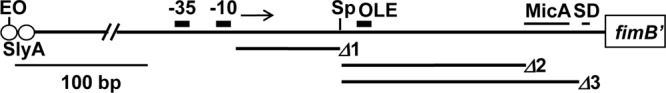
The organization of the *fimB* promoter and 5′ UTR. The extents of deletion mutations Δ1 to Δ3 are indicated by solid lines. Also indicated are the positions of the MicA target sequence in the *fimB* mRNA (26), the predicted *fimB* Shine-Dalgarno sequence (SD), and the *ops* site-like element OLE. The *fimB* promoter −35 and −10 regions (shaded rectangles), transcriptional start site and direction (arrow), and previously characterized SlyA binding sites *O_SA1_* and *O_SA2_* (34) are also shown. The start of the *fimB* ORF is indicated by the labeled box. Sp (SphI) and EO (EcoO109I) correspond to the restriction endonuclease sites used in this study. The ClaI site used lies within the *fimB* ORF further downstream than the region included in the diagram. The scale of the diagram (100 bp) is indicated by an additional horizontal line. The parallel diagonal lines denote that *O_SA1_* and *O_SA2_* lie further upstream of the *fimB* promoter than indicated by the linear scale of the diagram.

Direct control by RfaH requires a *cis*-acting *ops* (operon polarity suppressor; consensus of 5′ R GGCGGTAGYNT) site downstream of the transcriptional start site, typically positioned far upstream of the translational start site (35, 36). *fimB* has a large (271-bp) 5′ UTR, and to screen for *cis*-active sequences required for RfaH control, three deletions were constructed in this region and transferred into the chromosome at *fim* to replace the wild-type regulatory region of the FimB-LacZ fusion (Fig. 3). The first deletion (Δ1) extends from immediately adjacent to the −10 region of the *fimB* promoter (−277 bp to −203 bp relative to the *fimB* open reading frame [ORF]) to an SphI restriction endonuclease site. The second (Δ2) and third (Δ3) deletions extend from the SphI site to −58 bp and −20 bp relative to the *fimB* ORF, respectively. The best match (5′AAGGGA) to the consensus Shine-Dalgarno sequence (5′ AGGAGG) extends from −12 bp to −7 bp relative to the *fimB* ORF.

**Fig 4:**
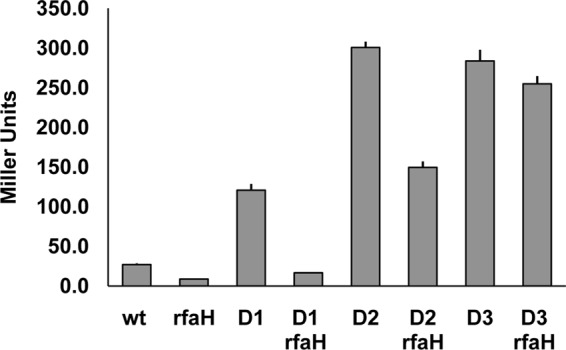
The effects of Δ*1* to Δ*3* mutations on the β-galactosidase produced by a FimB-LacZ fusion. The wild-type (wt) and mutant strains indicated were grown and processed as described in Materials and Methods.

All of the deletion mutations increased the expression of the FimB-LacZ fusion to a greater or lesser extent, suggesting that the long intergenic region of *fimB* has a detrimental effect on *fimB* expression overall (Fig. 4). While the Δ1 mutation increased the response to RfaH considerably (3-fold in the wild type to 7-fold in the mutant), the Δ2 mutation decreased the response to RfaH to 2-fold and the Δ3 mutation eliminated it almost entirely.

### Effect of RfaH on MicA inhibition of *fimB* expression.

**Fig 5:**
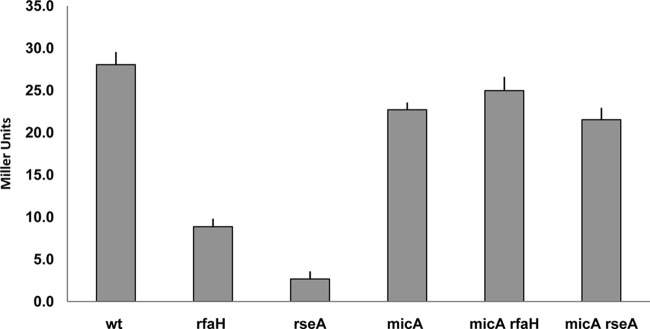
The effects of *micA*, *rfaH*, and *micA rfaH* double mutations on the β-galactosidase produced by a FimB-LacZ fusion. The wild-type (wt) and mutant strains indicated were grown and processed as described in Materials and Methods, except that the growth medium used contained 1 mM nicotinic acid to allow growth of the *rseA* mutants which contain a linked *nadB*::Tn*10* mutation.

The σ^E^-controlled regulatory sRNA MicA inhibits *fimB* expression (26). The mRNA binding target for MicA lies immediately upstream of the *fimB* Shine-Dalgarno sequence (extending from −9 to −46) and is thus conserved in its entirety in the Δ2 mutation but absent from the Δ3 mutation (Fig. 3). These observations suggested to us that RfaH might activate *fimB* expression by somehow preventing MicA inhibition. Although MicA surprisingly had a net stimulatory effect on *fimB* expression in the wild-type background, mutation of *micA* nevertheless suppressed the stimulatory effect of RfaH on *fimB* expression entirely (Fig. 5). As a control, the effect of RseA on *fimB* expression was also tested. RseA prevents induction of the σ^E^ regulon by sequestering the sigma factor to the inner membrane (37). As expected, *fimB* expression was inhibited strongly in the *rseA* mutant background and this effect was also suppressed in an *rseA micA* double mutant. The results of these experiments thus support the conclusion that RfaH somehow prevents MicA inhibition of *fimB* expression.

### Loss of RfaH induces *micA* and *rpoH*P3 expression.

**Fig 6:**
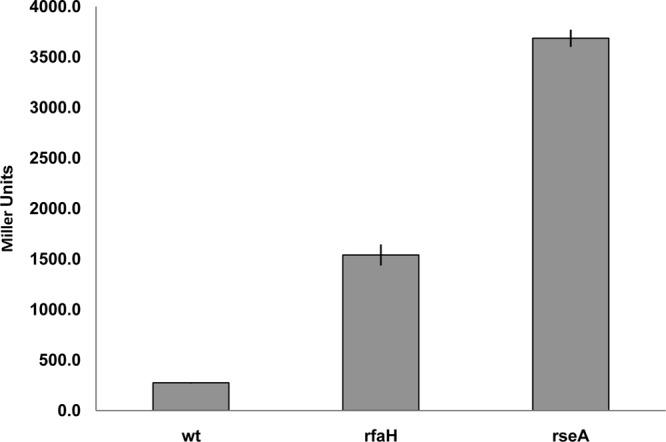
The effects of *rfaH* and *rseA* on the β-galactosidase produced by a *micA-lacZ* fusion. The wild-type (wt) and mutant strains indicated were grown and processed as described in Materials and Methods, except that the growth medium used contained 1 mM nicotinic acid to allow growth of the *rseA* mutants which contain a linked *nadB*::Tn*10* mutation.

The results described above suggested that RfaH might exert an indirect effect on *fimB* expression by controlling *micA* expression. To test this hypothesis, the effect of both RfaH and RseA on expression of a *micA-lacZ* transcriptional fusion was determined. The results of this experiment (Fig. 6) demonstrate that loss of RfaH results in the induction of *micA* transcription, albeit to a lesser extent than that observed in the *rseA* mutant background (>5-fold versus >13-fold).

**Fig 7:**
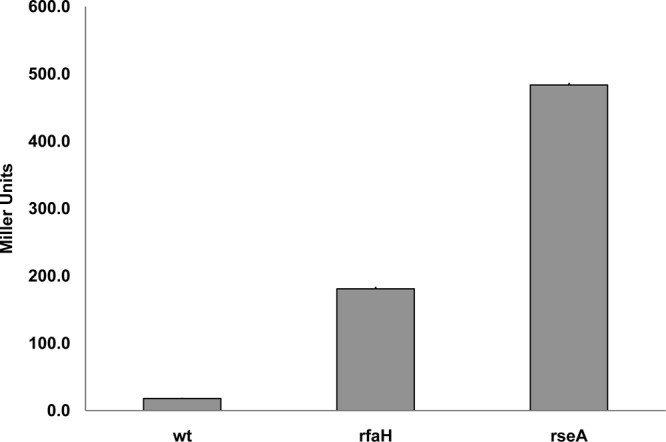
The effect of *rfaH* and *rseA* on the β-galactosidase produced by a *rpoH*P3::*lacZ* fusion. The strains indicated were grown and processed as described in Materials and Methods, except that the growth medium used contained 1 mM nicotinic acid to allow growth of the *rseA* mutants which contain a linked *nadB*::Tn*10* mutation.

We supposed either that RfaH could activate *micA* expression directly or that, more likely, its loss leads to induction of the σ^E^ regulon in general. To distinguish between these possibilities, the effect of RfaH on expression of the σ^E^-specific *rpoH*P3 promoter (38) was also tested (Fig. 7). As expected, expression of the *rpoH*P3-*lacZ* construct was induced significantly by mutation of *rseA* (27-fold). Mutation of *rfaH* had a smaller (10-fold) effect, consistent with the more modest effect of RfaH on both *fimB* and *micA* expression.

Notwithstanding the results described above, it still seemed possible that RfaH might also exert a direct effect on *fimB* expression by binding to an *ops*-like element in the *fimB* 5′ UTR. This seemed plausible because the Δ2 mutation, which removes a significant part of the 5′ UTR, diminished the effect of RfaH on *fimB* expression (Fig. 4). Moreover, a search of the 5′ UTR of *fimB* highlighted a potential *ops*-like site (5′ TGGCGTTTGTAT; non-*ops*-matching bases underlined) positioned around 180 bp upstream of the *fimB* translational start (Fig. 3). This *ops*-like sequence (here termed OLE for *o**ps*-like element) lies 8 bp downstream of the SphI site present in the *fimB* 5′ UTR and hence is deleted in both the Δ2 and Δ3 mutants. However, the effect of *rseA* on *fimB* expression was also decreased from >10-fold in the wild-type background to <4-fold in the Δ2 mutant background, suggesting that the Δ2 mutation diminishes the effect of MicA on *fimB* expression (data not shown). Moreover, while mutation of OLE from 5′ TGGCGTTTGTAT to TCCGCTATCAAT did decrease *fimB* expression >8-fold, this effect did not require RfaH (data not shown). Furthermore, the OLE mutation also decreased the expression of the *fimB-lacZ* transcription fusion (data not shown), which mutation of *rfaH* does not (Fig. 1). We thus conclude that loss of RfaH leads to induction of σ^E^, and hence *micA*, and that this effect accounts for most, if not all, of the stimulatory effect of RfaH on *fimB* expression.

### The effect of procaine on *fimB* expression and FimB recombination.

**Fig 8:**
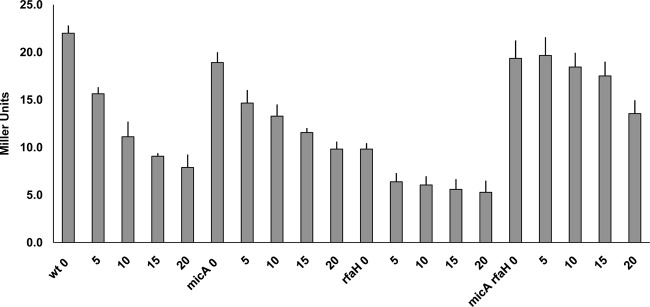
The effects of *micA*, *rfaH*, and *micA rfaH* double mutations on the β-galactosidase produced by a FimB-LacZ fusion in the presence and absence of procaine. Procaine was included at the concentrations (0 to 20 mM) specified. The wild-type (wt) and mutant strains indicated were grown and processed as described in Materials and Methods.

**Fig 9:**
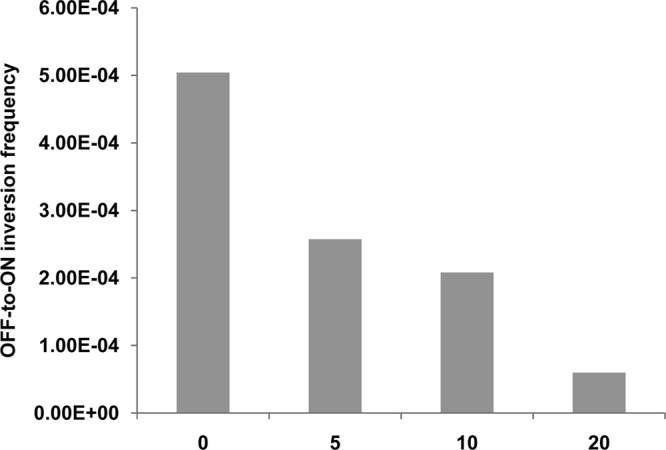
The effects of procaine on FimB off-to-on recombination per cell per generation. Procaine was included at the concentrations (0 to 20 mM) specified. The wild-type strain was grown and processed as described in Materials and Methods. The values shown are the means of at least five measurements.

σ^E^, and hence MicA, is induced by procaine and by ethanol (39, 40). As noted above, although MicA had a net stimulatory effect on *fimB* expression under noninducing conditions (Fig. 5), inclusion of increasing amounts of procaine (Fig. 8) or of ethanol (data not shown) led to a dose-dependent decrease in *fimB* expression as expected. FimB recombination was also inhibited by procaine as anticipated (Fig. 9). Procaine induces σ^E^ by activating the EnvZ-OmpR two-component regulatory system (39). In agreement with this assertion, the effect of procaine on *fimB* expression was also diminished in an *ompR* mutant background. Moreover, the level of *fimB* expression in a *micA ompR* double mutant was indistinguishable from that in the *ompR* single mutant background (data not shown).

**Fig 10:**
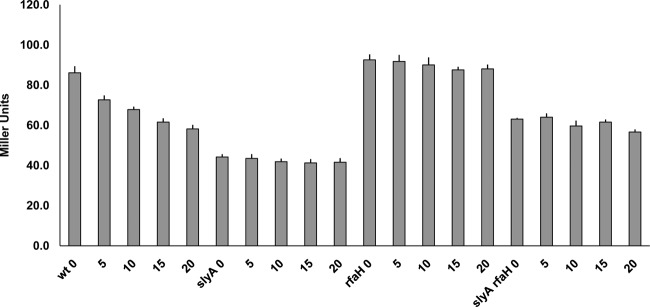
The effects of *rfaH* and *slyA* mutations on the β-galactosidase produced by a *fimB-lacZ* transcriptional fusion in the presence and absence of procaine. Procaine was included at the concentrations (0 to 20 mM) specified. The wild-type (wt) and mutant strains indicated were grown and processed as described in Materials and Methods.

Surprisingly, inhibition of *fimB* expression by both procaine (Fig. 8) and ethanol (data not shown) was still apparent, albeit to a decreased extent, in the *micA* mutant background. Unexpectedly, RfaH actually inhibited *fimB* expression in the absence of MicA under σ^E^-inducing conditions. Further work demonstrated that procaine also inhibits expression of the *fimB-lacZ* transcriptional fusion and that this effect requires RfaH (Fig. 10) but not OmpR (data not shown).

The results described above suggest that procaine might trigger an alternative envelope stress response pathway that is also controlled by RfaH. According to this scenario, we supposed that both procaine and RfaH activate the pathway and that the cognate regulator of the system inhibits *fimB* transcription. It has been shown previously that *fimB* transcription is controlled by the response regulator RcsB that forms part of the Rcs phosphorelay system, a regulatory pathway that is also responsive to envelope stress (41). However, it was found that *fimB* transcription was unaffected by RcsB under the growth conditions used in this study and that the effect of procaine on the expression of the *fimB-lacZ* transcriptional fusion remained intact in this mutant background (data not shown). In contrast, the response of *fimB* transcription to procaine was found to be dependent upon SlyA (Fig. 10). Furthermore, the inhibitory effect of RfaH on *fimB* transcription was enhanced in the *slyA* mutant background.

The results presented above are consistent with our model that RfaH activates *fimB* expression by preventing MicA inhibition. However, they also suggest that RfaH can, at least in the presence of procaine, somehow inhibit *fimB* transcription by a mechanism that involves neither OmpR nor RcsB but which does require SlyA. These results highlight the complexity of the RfaH regulatory circuit that controls *fimB* expression.

## DISCUSSION

RfaH-binding *ops* elements are characteristically found in long 5′ UTRs, far upstream of ORFs. Moreover, UPEC mutants lacking *rfaH* are attenuated for virulence in an ascending mouse model of urinary tract infection (21). Since *fimB* has a relatively large (271-bp) 5′ UTR and type 1 fimbriation is a virulence factor in the mouse model, we considered it possible that RfaH is an activator of *fimB* expression. Here, we demonstrate that RfaH does indeed enhance *fimB* expression, but further analysis reveals that this effect is indirect.

**Fig 11:**
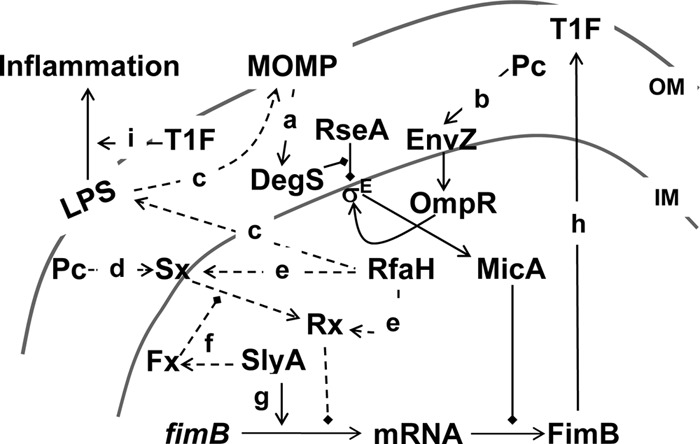
Model for the control of *fimB* expression and type 1 fimbriation by RfaH and MicA. (a) Misfolded outer membrane proteins (MOMP) activate protease DegS to cleave RseA, releasing σ^E^ to activate *micA* transcription (37, 46). (b) Procaine (Pc) activates EnvZ/OmpR to also induce σ^E^ and hence *micA* expression (39). (c) Mutation of *rfaH* also leads to changes in LPS core biosynthesis that cause misfolding of outer membrane proteins and hence induction of σ^E^ and hence *micA* expression. (d) Procaine activates an alternative envelope stress pathway (Sx-Rx). (e) RfaH is postulated to inhibit *fimB* transcription indirectly by activation expression of Sx and/or Rx. (f and g) SlyA enhances *fimB* expression directly (34) (f) but is also postulated (g) to enhance expression of an unknown factor (Fx) that prevents Sx-Rx signaling in the absence of procaine. (h) FimB catalyzes off-to-on inversion of *fimS* to enhance expression of type 1 fimbriae (T1F). (i) Type 1 fimbriae stimulate inflammation by enhancing LPS-activated host signaling pathways (10–12). Stimulatory and inhibitory interactions are indicated by arrows and diamonds, respectively. Dotted lines represent speculative pathways. OM, outer membrane; IM, inner membrane.

In support of the hypothesis that RfaH enhances *fimB* expression, it was found in an initial experiment that expression of a FimB-LacZ protein fusion was diminished around 3-fold in an *rfaH* deletion mutant. Surprisingly, however, expression of a comparable *fimB-lacZ* transcriptional fusion was elevated slightly in the absence of RfaH, indicating that RfaH activates *fimB* expression selectively at the posttranscriptional level. Moreover, deletion analysis of the *fimB* 5′ UTR indicated that, rather than requiring sequences far upstream of the *fimB* ORF, RfaH control is dependent upon sequences close to the ribosome binding site. Further work demonstrates that RfaH activates *fimB* expression indirectly by controlling induction of σ^E^ and hence the sRNA MicA (Fig. 11).

In addition to controlling the expression of a number of virulence factors, RfaH enhances expression of the *waaQ* operon required for LPS core biosynthesis (15, 16). Alterations to LPS core biosynthesis, apparently by inducing misfolding of outer membrane proteins, can also induce σ^E^ (42). While not proven here, we postulate that induction of σ^E^ in the *rfaH* mutant reflects the involvement of RfaH in LPS biosynthesis (Fig. 11). We note that the increased autoaggregation factor antigen 43 (Ag43)-enhanced biofilm formation observed in an *rfaH* mutant background was also attributed in part to changes in LPS biosynthesis (17).

The effect of procaine on σ^E^ induction, and hence *micA* expression, was reported previously to be dependent upon OmpR (39). Our results agree with this since the effect of procaine on *fimB* expression was diminished in an *ompR* mutant and the level of *fimB* expression in the *micA ompR* double mutant was indistinguishable from that of the *ompR* single mutant across the range of procaine levels (0 to 20 mM) tested (data not shown). Surprisingly, the residual effect of procaine on *fimB* expression in a *micA* mutant is largely dependent on RfaH (Fig. 8). Further work shows that procaine inhibits expression of the *fimB-lacZ* transcriptional fusion as well and that this effect requires RfaH (Fig. 10) but not OmpR (data not shown). We suppose that this additional effect of procaine involves an alternative stress-response pathway and an unidentified transcriptional repressor (Rx) (Fig. 11). RfaH has also been shown somehow to inhibit transcription of *flu*, which encodes Ag43 (17). This effect, which is due neither to changes in *flu* phase variation *per se* nor to altered control by known regulator OxyR or Dam, suggests that *fimB* and *flu* transcription may both be repressed by Rx.

The effect of RfaH and procaine on *fimB* transcription is not dependent upon OLE (data not shown). Moreover, we have also ruled out involvement of the Rcs phosphorelay system, which has been reported to control *fimB* expression and which is also responsive to envelope stress (data not shown) (41). On the other hand, the effect of procaine on *fimB* transcription requires SlyA (Fig. 10). Although these results are open to interpretation, we favor a model in which RfaH is necessary for expression of the alternative stress-response pathway while loss of SlyA leads to its constitutive activation (Fig. 11). We suppose that SlyA activates the expression of an additional unidentified factor (Fx) that somehow alters the signaling pathway to make it responsive to procaine (Fig. 11).

Type 1 fimbriae, which are anchored in the bacterial outer membrane, facilitate the delivery of LPS to the TLR4 signaling pathway in CD14-negative epithelial cells (10). Furthermore, in contrast to phase-locked-off bacteria, MicA inhibits *fimB* expression in phase-locked-on fimbriate cells even in the absence of exogenous inducers of σ^E^ (our unpublished data). This suggests to us that fimbrial biosynthesis itself imposes significant stress on the outer membrane, as has been reported for other outer membrane proteins (38). We thus propose that suppressing *fimB* expression, and hence type 1 fimbriation, in response to the integrity of the bacterial outer membrane is an adaptation that enhances bacterial survival both by diminishing envelope stress and by limiting the host inflammatory response. The fact that RybB, a second σ^E^-dependent sRNA, inhibits *fimA* expression would provide an additional mechanism to limit fimbrial expression in phase-on bacteria (26). Indeed, we suppose that this explains why nonfimbriate cells are produced even when *fimS* is locked in the on phase, or when *fimS* is replaced with the isopropyl-β-d-galactopyranoside (IPTG)-inducible *tac* promoter (43). Mutation of *surA* also induces σ^E^ (44), and as expected, we have found that *fimB* expression was also decreased in a *surA* mutant (data not shown). While SurA enhances the correct localization of the *fim* usher (FimD) to the outer membrane, our results indicate that decreased *fimB* expression, and hence off-to-on inversion of *fimS*, as well as induction of *rybB*, also contributed to diminished type 1 fimbriation observed in a *surA* mutant (45).

A BLAST search of the nucleotides of the *fimB* mRNA predicted to bind to MicA demonstrates that these sequences are perfectly conserved in all of the E. coli strains for which DNA sequence data are available. Thus, the regulation of *fimB* expression by MicA, and its control by factors such as RfaH and SurA, is likely to have general significance for the control of type 1 fimbriation in most, if not all, E. coli strains. Further work will determine how RfaH controls σ^E^, as well as the response of *fimB* transcription to procaine, and the full extent of these control pathways on type 1 fimbriation in commensal and pathogenic strains alike.

## Supplementary Material

Supplemental material
