# Supplementary material for: RfaH Suppresses Small RNA MicA Inhibition of fimB Expression in Escherichia coli K-12
Source: J Bacteriol. 2014 Jan;196(1):148–56. doi: 10.1128/JB.00912-13 (PMC3911127; doi:10.1128/JB.00912-13)
Supplement: Supplemental material [file supp_196_1_148__index.html]

Supplemental material 

# RfaH Suppresses Small RNA MicA Inhibition of *fimB* Expression in Escherichia coli K-12

## Supplemental material

**Files in this Data Supplement:**

- Supplemental file 1 -

  Table S1, oligonucleotides

  PDF, 76K
