# Supplementary material for: RfaH Suppresses Small RNA MicA Inhibition of fimB Expression in Escherichia coli K-12
Source: J Bacteriol. 2014 Jan;196(1):148–56. doi: 10.1128/JB.00912-13 (PMC3911127; doi:10.1128/JB.00912-13)
Supplement: Supplemental material [file JB.00912-13_zjb999092961so1.pdf]

Oligonucleotides used in this study

Construct	Name	Oligonucleotides (5’-3’)
*Δ*1	Site 6F 5’	GAGTCTAAACAAGGGGAGC
	P2D1Rev	GGGGGCATGCTATATATCATGTGATATGTTC
*Δ*2	5’UTR-sml-Del	GGGGGGGCATGCTGGATTATTGCTAACCCAGCACAG
	ClaI-Umut	CCCCCCATCGATGGATATATATACACTTTGCC
*Δ*3	DelSphI-RBS	GGGGGGGCATGCTAATTATAAGGGAAAAACGATGAAG x ClaI-Umut
OLE	RfaH-PutOP-fw	GGGGGGCATGCAAGAGCATCCGCTATCAATGGCAACGTTATTATAATTAACAG x ClaI-Umut
rfaH complement	rfaH-lacUV5-new	GGGGGGATCCTCTTAATCTGCGCCCATTATACGGCG
	lacUV5-rfaH_down	GGGGAAGCTTGGCTGGCTGCCACCACGGATG
rfaH allele check	rfaH_fw	GCCTGGCACCGATGATCACCC
	rfaH_rv	GGAGCGAGCCTCACCATCGGG
Sequencing OLE	SphI-fw	GTCATATGTGAGACGGCTAGTTGAACG x ClaI-Umut
OLE transcriptional fusion	Ops-transcript	GGGGGATCCCCTAGTCTAGACTAGCGATGGATATATATACACTTTGCCTTAAGATCAATATCCG x RfaH-PutOP-fw
micA-lacZYA upstream	micA-up-xbaI	GGGGTCTAGAATAACAAATGCGCGTCTTTCATATACTCAGACTCGCCTG
	HindIII-luxS	GGGGAAGCTTTACGCGAACGCTGGCGG
micA-lacZYA downstream	micA-down-xbaI	GGGGTCTAGACATCATCCCTGAATTCAGAGATGAAATTTTGG
	KpnI-gshA	GGGGGGTACCGATCTTTACGAGTACGTAGCGGG
micA-lacZYA	Lac-	GGGGAAGCTTACGGGCAGACATGGCCTGCCCGG

orientation check of *lacZYA*	(HindIII)-down	x HindIII-luxS
	lacZ-up-PstI-AM	GGGGGGCTGCAGCCCACAGCCGCCAGTTCCGC x KpnI-gshA
